# A Versatile Method for Cell-Specific Profiling of Translated mRNAs in *Drosophila*

**DOI:** 10.1371/journal.pone.0040276

**Published:** 2012-07-06

**Authors:** Amanda Thomas, Pei-Jung Lee, Justin E. Dalton, Krystle J. Nomie, Loredana Stoica, Mauro Costa-Mattioli, Peter Chang, Sergey Nuzhdin, Michelle N. Arbeitman, Herman A. Dierick

**Affiliations:** 1 Department of Molecular & Human Genetics, Baylor College of Medicine, Houston, Texas, United States of America; 2 Department of Biomedical Sciences, College of Medicine, Florida State University, Tallahassee, Florida, United States of America; 3 Department of Neuroscience, Baylor College of Medicine, Houston, Texas, United States of America; 4 Department of Molecular & Cellular Biology, Baylor College of Medicine, Houston, Texas, United States of America; 5 Program in Developmental Biology, Baylor College of Medicine, Houston, Texas, United States of America; 6 Section of Molecular and Computational Biology, Department of Biological Sciences, University of Southern California, Los Angeles, California, United States of America; 7 Department of Pathology and Immunology, Baylor College of Medicine, Houston, Texas, United States of America; University of Houston, United States of America

## Abstract

In *Drosophila melanogaster* few methods exist to perform rapid cell-type or tissue-specific expression profiling. A translating ribosome affinity purification (TRAP) method to profile actively translated mRNAs has been developed for use in a number of multicellular organisms although it has only been implemented to examine limited sets of cell- or tissue-types in these organisms. We have adapted the TRAP method for use in the versatile *GAL4/UAS* system of *Drosophila* allowing profiling of almost any tissue/cell-type with a single genetic cross. We created transgenic strains expressing a GFP-tagged ribosomal protein, RpL10A, under the control of the *UAS* promoter to perform cell-type specific translatome profiling. The GFP::RpL10A fusion protein incorporates efficiently into ribosomes and polysomes. Polysome affinity purification strongly enriches mRNAs from expected genes in the targeted tissues with sufficient sensitivity to analyze expression in small cell populations. This method can be used to determine the unique translatome profiles in different cell-types under varied physiological, pharmacological and pathological conditions.

## Introduction

*Drosophila melanogaster* is a powerful, fast, tractable, and highly effective model system to dissect gene function *in vivo* to understand fundamental biological processes. Despite the plethora of molecular-genetic tools available in flies [1]–[3], it is still difficult to examine gene expression in a tissue-specific manner, let alone in small cell populations. Tissue- and cell-type specific expression profiling is nevertheless important to understand the biology of particular cell types and to uncover the downstream effect of single gene mutations in a spatial/temporal specific manner, especially with respect to cell autonomous and non-autonomous effects of genes and their mutations. Whole animal or even body-part-specific expression studies have important limitations. For example, in *Drosophila*, a significantly higher percentage of transcripts are identified when gene expression is examined in a tissue-specific manner compared to the entire organism [4]. This suggests that many rare or tissue-specific transcripts are not detected when the whole organism or large body parts are used as starting material for these expression studies. Currently, most tissue-specific transcriptome analyses in *Drosophila* are time consuming and rely on dissection techniques, leading to variability because of dissecting irregularities and small sample sizes. This can result in unwanted detection of transcripts from other tissue-types and under representation of rare transcripts. Moreover, some tissues cannot be dissected and cell-specific analyses are not possible.

In *Drosophila*, two transgenic methods have been developed to analyze the transcriptome. Both of these methods are integrated into the binary *GAL4/UAS* system [5] and thus allow profiling in a tissue/cell-type specific manner. The first method is based on transgenic expression of an epitope-tagged human or *Drosophila* polyA binding protein (PABP) from a *UAS* promoter, and has been used to capture and enrich eye specific mRNAs, although paradoxically driving expression of this transgene in the eye squelches expression of some eye specific genes [6]. The second method, called TU tagging, is based on transgenic expression of *Toxoplasma gondii* phosphoribosyl transferase (UPRT) from a *UAS* promoter, which allows for tissue-specific incorporation of 4-thiouracil (TU) into newly synthetised mRNA, when TU is fed to the adult flies or larvae [7]. After RNA isolation from the animals, only the mRNAs that have incorporated TU are coupled to biotin via the thiol-containing nucleotide and purified using streptavidin-coated beads [7]. In our unpublished studies, we found that TU feeding can lead to background incorporation into mRNA and is toxic to flies. A third non-transgenic method is based on manual isolation of GFP positive cells [8], which is labor-intensive and hard to implement for high-throughput purposes. In addition, none of these methods uniquely profile the cell or tissue *translatome*, consisting of the actively translated mRNAs that are likely the most important messages for the immediate activity changes occurring in cells.

In mice, a transgenic method was developed to isolate polysome-associated mRNA from specific brain regions and different neuronal cell types [9]–[10]. Using BAC transgenics, the green fluorescent protein (GFP) was fused to the N-terminus of the large-subunit ribosomal protein L10a (RpL10a) and expressed in specific neuronal populations [10]. The GFP tagged polysomes were subsequently affinity purified to isolate translated mRNAs from these neuronal populations. A similar method has also been used in several other species to profile the translatome from specific tissues [11]–[14].

In this study, we have adapted this translating ribosome affinity purification (TRAP) system to examine actively translated mRNAs in a cell-type specific manner for use in *Drosophila* with the versatile binary *GAL4/UAS* system [5]. We have generated transgenic strains expressing GFP tagged *Drosophila* RpL10A from a *UAS* promoter. We show that this tagged RpL10A fusion protein is efficiently incorporated into ribosomes and polysomes. We expressed the *UAS-GFP::RpL10A* transgene in neurons using a pan-neuronal driver and sequenced the neuronal translatome from adult heads of these flies. We compared the affinity purified neuronal mRNAs to mRNAs derived from whole heads and found strong enrichment of mRNAs encoded by genes with known neuronal expression and strong depletion of mRNAs known to be expressed in non-neuronal head tissues. We also captured translated mRNAs from a small cell population of neurosecretory cells in the adult brain and strongly enriched mRNAs encoding a neuropeptide expressed in these cells while strongly depleting mRNAs encoding a neuropeptide that is not expressed in these cells, showing that this method can be used to profile small cell populations. Our data indicate that we have developed a powerful method to profile the translatome of any cell population for which a Gal4 driver strain exists and further strengthens the impressive repertoire of reagents that can be used to study the pomace fly *Drosophila melanogaster*.

## Results

### Generation of a *GAL4/UAS* TRAP Method

**Figure 1:**
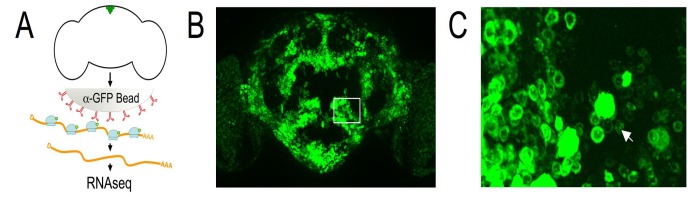
Polysome affinity purification strategy using *GAL4/UAS-GFP::RpL10A*. (A) Schematic representation of the polysome affinity purification method from adult *Drosophila* brains expressing GFP-tagged RpL10A in a small population of neurons. Lysates from heads of transgenic animals are incubated with beads (shaded grey) coated with GFP antibodies (red). Ribosomes (light blue) associated along the actively translated mRNA strands (orange) are captured on the beads and washed, followed by an RNA extraction step. RNA can then be used for qRT-PCR or purified for sequencing. (B) Live image of an adult brain from a fly expressing GFP-tagged RpL10A in all neurons (*Elav-GAL4>UAS-GFP::RpL10A*). The GFP expression pattern is consistent with the *GAL4* driver. (C) Enlarged view of a section of the brain in (B), showing that GFP localization is predominantly perinuclear and nucleolar (white arrow).

We have adapted the translating ribosome affinity purification (TRAP) methodology to examine actively translated mRNAs in a cell-type specific manner for use with the versatile binary *GAL4/UAS* system [5](**Figure 1A**). We generated transgenic strains, each containing a random insertion of *UAS-GFP::RpL10A* (**Figure S1**), thus providing an inducible and cell-specific method for the expression of tagged ribosomal subunits and limited only by the availability of *GAL4* expression lines. We generated 16 independent insertions and several showed strong expression in the brain when crossed to the pan-neuronal driver *Elav-GAL4* [15](**Figure 1B** **& Table S1**). The GFP-tagged RpL10A protein was predominantly localized in the cytoplasm around the nucleus and in the nucleolus (**Figure 1C** **& Figure S2**), consistent with the localization of endogenous ribosomal proteins and mammalian GFP-tagged RpL10a [9]. Most of the insertion lines are homozygous viable and generate viable flies when crossed to a ubiquitous or a pan-neuronal driver. In addition, we found that the flies showed no developmental delay and appeared healthy (**Table S1**), although we have not examined all tissues in this study.

### *GAL4* Driven *UAS-GFP::RpL10A* Incorporates into Ribosomes and Polysomes

**Figure 2:**
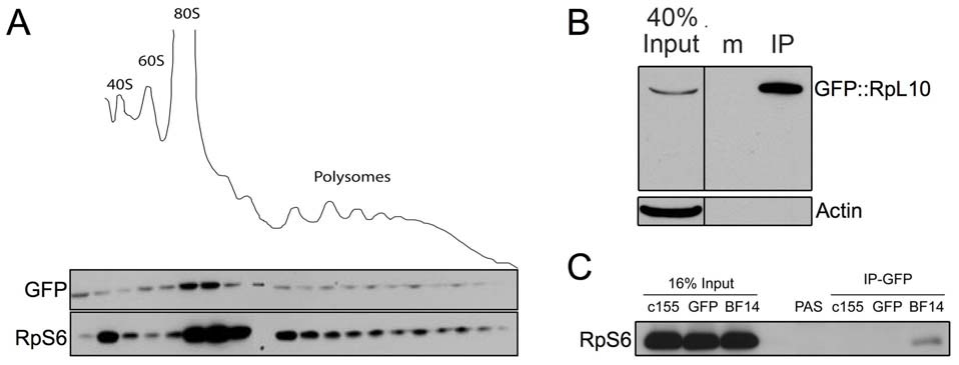
Polysome incorporation of GFP tagged RpL10A. (A) Sucrose gradient polysome fractionation from heads of *Elav-GAL4>UAS-GFP::RpL10A* flies shows the different ribosomal and polysomal fractions. Protein extracts were run on Western blots and probed with GFP and RpS6 antibodies, showing that the GFP-tagged RpL10A displays qualitatively similar incorporation into the polysomes but not into the small 40S ribosomal unit. (B) Immunoprecipitation from lysted made from 50 adult heads of *Elav-GAL4>UAS-GFP::RpL10A* flies following polysome immunoprecipitation with PAS beads coated with GFP antibodies (IP) or mouse IgG antibodies as a mock control (m). Lysate from the input fraction without the immunoprecipitation step was also loaded on the gel. The GFP-tagged fusion protein is efficiently precipitated from the GFP coated beads but not from the mock coated beads. Actin is only present in the unprecipitated lysate and not in the IP fraction. (C) Western blot of lysates from heads of the *Elav-GAL4* (c155) strain and flies expressing GFP or GFP-tagged RpL10A (strain BF14; Table S1) with the *Elav-GAL4* driver. All total lysates (16% input) show strong signal for the small ribosomal protein RpS6. After polysome affinity purification, only the immunoprecipitate from *Elav-GAL4>UAS-GFP::RpL10A* flies (strain BF14) show staining for RpS6, showing that whole ribosomes are precipitated.

We next examined whether GFP-tagged RpL10A incorporates into assembled ribosomes and polysomes. To do so, we performed sucrose gradient centrifugation [16] followed by Western blotting on extracts from heads of *Elav-GAL4>UAS-GFP::RpL10A* flies. As expected, we found GFP-tagged RpL10A in the large ribosomal subunit, the monosome fraction, and the polysome fractions (**Figure 2A**). To assess incorporation levels, we compared the levels of RpS6, RpL10 and GFP::RpL10A in each fraction normalized to the signal intensity of the input fraction. We estimate that the signal intensity of the tagged protein in the fractions ranged between 10% and 30% of the signal of endogenous RpL10 (**Figure S3**), demonstrating that the GFP-tagged RpL10A variant is incorporated into a portion of polysomes without a bias to a particular polysome fraction. We also analyzed the immunoprecipitated polysome complexes by Western blot and found strong enrichment of GFP-tagged RpL10A as compared to the input lysate, and we found no signal in immunoprecipitates when we used non-specific antibodies (**Figure 2B**). In addition, we detected RpS6 in the immunoprecipitate of *Elav-GAL4>GFP::RpL10A* flies, but not in controls (*Elav-GAL4* [*c155*] and *Elav-GAL4>UAS-GFP* [*GFP*]) (**Figure 2C**). These results demonstrate that GFP-tagged RpL10A incorporates into the large ribosomal subunit and that monosomes and polysomes can be immunoprecipitated from head extracts using the tagged transgenic RpL10A.

### Polysome Complexes Contain mRNAs

**Figure 3:**
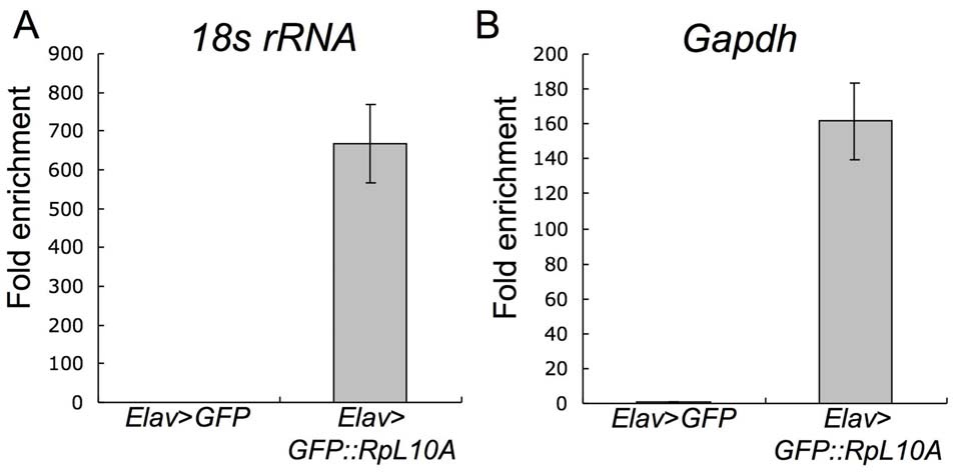
Enrichment of *18S* rRNA and *Gapdh* mRNA from neuronal ribosomes and polysomes. Total RNA was extracted from lysted from 50 adult heads of *Elav-GAL4>UAS-GFP::RpL10A* compared to *Elav-GAL4>GFP* flies following immunoprecipitation with PAS beads coated with GFP antibodies. The RNA samples were reverse transcribed and amplified with *18S* rRNA primers (A) or *Gapdh* primers (B). Fold enrichment of RNA was calculated in the *Elav-GAL4>UAS-GFP::RpL10A* compared to *Elav-GAL4>GFP* samples, set at 1x. Data are means +/− S.E.M. averaged from three replicates from 2 biological repeats.

To determine whether we could isolate and enrich RNA from these polysome complexes, we extracted total RNA from the immunoprecipitated polysome complexes. Using qRT-PCR we found more than 600 and 150 fold enrichment of *18 S* rRNA and *Gapdh* mRNA in the affinity purified polysomes from *Elav-GAL4>UAS-GFP::RpL10A* heads as compared to *Elav-GAL4>UAS-GFP* controls (**Figure 3**).

### The Adult Neuronal Translatome Profile

To further test whether we could fully profile and enrich for the whole translatome from neurons of the head, we extracted mRNA from the immunoprecipitated polysome fraction from *Elav-GAL4>GFP::RpL10A* heads and from wild-type *CantonS* heads, and we made libraries for Illumina RNA sequencing [17]. We found strong enrichment of transcripts from genes with known neuronal expression in the polysome purified mRNAs as compared to the mRNA derived from whole head extract (**Table S2**). Half of the top 40 enriched transcripts are from neuropeptide-encoding genes that are enriched 12 to 70 fold compared to whole head mRNA extracts (**Table S2**). Of the top 20 enriched transcripts the majority are from genes with known neuronal expression and were otherwise underrepresented in whole head mRNA (**Table 1**). Four of these enriched transcripts are uncharacterized and their enrichment in neurons suggests that they play a role in the brain of the fly, further demonstrating that this approach can lead to novel discovery of gene function. We also analyzed transcripts that were depleted in the neuronal translatome and found that many of these transcripts are encoded by genes that are not highly expressed in neurons. Furthermore, we analyzed the levels of transcripts encoded by three groups of genes that we expect to be depleted in neuronal populations, as they had previously been identified as enriched in glia [18], fat body [19] and cuticle [20]. We found that approximately 60% of these genes were significantly depleted from our neuronal polysome sample (**Table S3, S4, S5**).

**Table 1:** Genes with the top 20 enriched transcripts in *Elav-GAL4>UAS-GFP::RpL10A* vs *CantonS* heads.

FlyBase ID	Gene Name	Whole Head Mean (fpkm)	Whole Head S.E.M	RpL10A IP Mean (fpkm)	RpL10A IP S.E.M	Fold enrichment
FBgn0259831	*CG34309*	2.01	0.46	349.02	64.17	173.25
FBgn0044050	*Ilp3*	14.72	1.19	1016.74	8.14	69.05
**FBgn0003227**	***rec***	**2.35**	**0.22**	**149.18**	**11.26**	**63.57**
FBgn0038343	*CG14871*	9.56	1.48	603.90	10.31	63.14
FBgn0052282	*dro4*	7.03	0.37	380.63	9.93	54.11
FBgn0034935	*CG13565*	11.74	1.43	628.30	20.10	53.54
FBgn0023178	*Pdf*	21.80	2.97	1043.43	3.58	47.86
FBgn0027109	*npf*	9.39	1.24	383.70	3.93	40.85
FBgn0036046	*Ilp2*	29.49	1.71	1054.50	14.99	35.76
FBgn0039722	*capa*	16.27	2.09	579.32	18.92	35.61
**FBgn0085452**	***CG34423***	**3.02**	**0.38**	**99.43**	**5.36**	**32.93**
FBgn0032336	*Ast-C*	40.41	4.42	1174.91	22.56	29.07
FBgn0028374	*hug*	22.81	2.57	652.03	12.20	28.58
FBgn0000500	*Dsk*	18.78	2.67	502.91	4.14	26.78
FBgn0036713	*Mip*	21.97	3.22	527.56	17.15	24.01
**FBgn0001223**	***Hsp22***	**3.18**	**0.17**	**73.28**	**3.31**	**23.03**
FBgn0034069	*CG8401*	4.52	0.35	96.03	7.54	21.23
FBgn0044048	*Ilp5*	9.20	0.67	194.14	2.47	21.10
FBgn0011581	*Dms*	91.62	11.11	1882.59	39.31	20.55
**FBgn0000564**	***Eh***	**2.34**	**0.49**	**45.88**	**3.15**	**19.59**

**Figure 4:**
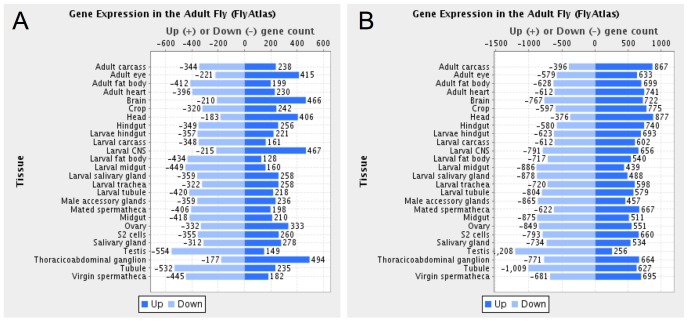
Tissue specific expression levels of significantly enriched or depleted genes in the neuronal translatome compared to whole head mRNA. Comparison of the lists of genes that have transcripts that are significantly and substantially enriched (A; 872 genes) or depleted (B; 1,755 genes) in the polysome-enriched fraction (q<0.05, >2-fold enriched or depleted, respectively) to Flyatlas microarray expression data. For this analysis we generated two lists of genes: those that are either significantly enriched or depleted in the polysome pull-down fraction relative to mRNA derived from whole heads. The two lists were uploaded to Flymine [21], which generated the graphs presented that shows the number of genes from each list for which the levels of expression are significantly high or low, in several tissues of the fly, according to FlyAtlas [4] microarray data analysis. The genes with transcripts enriched in the polysome-enriched fraction have a greater number of genes with high expression in neuronal tissues as compared to low expression (A; see adult brain, eye, larval CNS, thoracicoabdominal ganglion), and this is not seen in the polyosme-depleted fraction. In other tissues examined by Flyatlas, the polysome-enriched and -depleted fractions do not show this pattern of substantially more genes with high expression in that tissue.

A further analysis of the genes that had transcripts with significant and substantial enrichment in polysome fraction (q<0.05, >2-fold enrichment; 872 genes) demonstrates that a large fraction of these genes have been shown to have high expression in neuronal tissues. We analyzed the expression of the 872 genes to determine if these genes had high or low expression in twenty-six fly tissues, for which expression data has been compiled by Flyatlas [4] and assessed using Flymine [21] (**Figure 4A** **& Table S2**). The 872 genes with polysome-enriched transcripts had a tissue-pattern of having the largest number of genes with up-regulated expression in tissues enriched for neurons, including the dissected adult eye (415/872 genes up-regulated), brain (466/872 genes up-regulated), thoracicoabdominal ganglion (494/872 genes up-regulated), and larval central nervous system tissues (467/872 genes up-regulated) (**Figure 4A** **& Table S2**). In contrast, there are fewer genes with transcripts that are enriched in the neuronal polysome fraction that also have up-regulated expression in the testis (149/872 genes up-regulated) and the larval fat body (128/872 genes up-regulated), as well as nearly all of the other tissues examined **(** **Figure 4A** **)**. While there are genes in the polysome-enriched fraction that have up-regulated expression in other tissues, this could reflect the pleiotropic nature of gene function, with these genes having up-regulated expression in the nervous system, as well as other tissues. Additionally, for some genes transcript enrichment in the polysome-enriched fraction could reflect a situation where these genes are not highly expressed in the nervous system, but are highly translated.

In contrast, genes that had transcripts that were depleted from the polysome-purified sample (q<0.05, >2-fold depletion, 1754 genes) did not show the same distribution, but were more evenly distributed with high and low expression among all the tissues analyzed by Flyatlas [4], [21] (**Figure 4B**). The polysome-depleted fraction should contain genes that are expressed in adult head tissues that are not highly translated in the nervous system. As such, it makes sense that many of the genes that are depleted in the polysome-enriched neuronal fraction, as compared to whole head mRNA, have high expression in the adult head (877/1754 genes up-regulated), adult carcass (867/1754 up-regulated) and fat body (699/1754 genes up-regulated). Given that the genes that are depleted in the polysome fraction are more evenly distributed as having high and low expression in the tissues examined by Flyatlas, suggests that these genes may be generally more pleiotropic in nature. Taken together, there is a clear pattern, with genes that have transcripts enriched in the polysome fraction having tissue-specific differences in their expression levels, with tissues enriched for the nervous system having the largest number of up-regulated genes as compared to down-regulated genes **(** **Figure 4A** **)**, which is not seen in the polysome-depleted fraction **(** **Figure 4B** **)**.

### TRAPping Small Cell Populations

**Figure 5:**
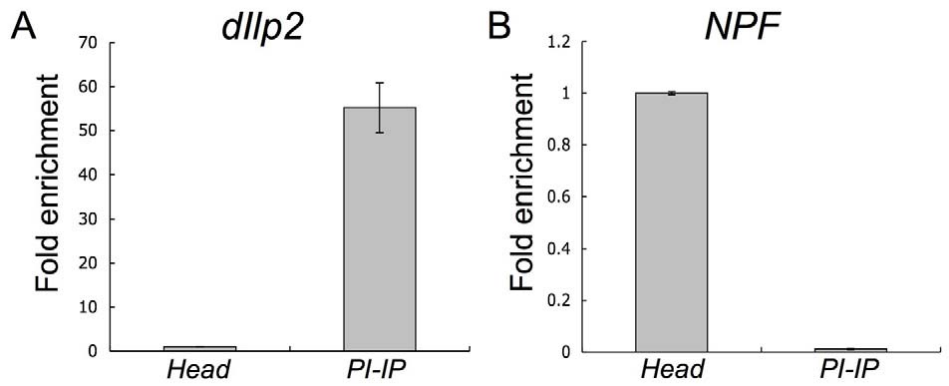
Enrichment of *dIlp2* and depletion of *NPF* from the *PI* translatome. qRT-PCR from total RNA extracts from affinity purified polysome fractions from neurons in the *pars intercerebralis* (from *50Y>UAS-GFP::RpL10A* flies) compared to whole head extracts from control flies. (A) *dIlp2* is expressed in *PI* neurons and shows ∼55 fold higher expression in the immunoprecipitate from *PI* neurons (*PI-IP*) as compared to whole head extract (*Head*). (B) NPF is not expressed in *PI* neurons and shows the opposite pattern: ∼80 fold lower in *PI-IP* than in whole head extract. Data are means +/− S.E.M. averaged from three replicates from 2 biological repeats.

We next tested whether this method is sensitive enough to profile small subpopulations of neurons. Using the *50Y* driver [22], we drove expression of *UAS-GFP::RpL10A* in the *pars intercerebralis* (*PI*), a small dorsomedial brain region of roughly 200 neurosecretory cells [23], [24] (**Figure S4**). We isolated RNA from the immunoprecipitated polysome complexes and compared expression to RNA from whole heads. Expression analysis of *dIlp2*, a gene expressed in only seven *PI* neurons [25], showed ∼55 fold enrichment in the polysome affinity purified sample from *PI* neurons (**Figure 5A**). In contrast, *NPF* mRNA, which is not expressed in *PI* neurons, was ∼80 fold depleted (**Figure 5B**).

## Discussion

In this study we generated transgenic lines carrying *UAS-GFP::RpL10A* that can be crossed to the large collection of strains that express *GAL4* in tissues/cells of interest to extract mRNAs associated with polysomes to enable efficient translatome profiling. Our results show that we can enrich for translated transcripts that are expressed in very small sets of cells, such as the PI neurons, as well as examine the full translatome in neurons, by coupling the TRAP method with next-generation sequencing approaches. There are currently two transgenic methods in *Drosophila* to perform tissue specific expression analysis [6]–[7], but neither has become broadly used due to various technical issues. Recently, specific cell-types from the adult brain were profiled by hand-collecting disassociated GFP expressing neurons [8]. This method was effective but labor intensive and so it is not practical for many biological questions. None of the above-discussed methods [6]–[8] specifically target polysome-associated mRNAs, which are the actively translated messages that are affecting the activity of a cell through the production of protein. The tool we desrcibe here to perform cell-specific translatome profiling will allow for the first time to address several questions regarding the life-cycle of mRNA molecules in *Drosophila*. Our results show that genes with the most abundant transcripts in the nervous system are not necessarily those that are present at the highest level in the ribosome and suggest that an understanding of which mRNAs are actively translated, coupled with transcriptome studies, will aid in a deeper understanding of gene function. In addition, this technique will solve problems of reduced specificity and sensitivity associated with transriptome analysis on whole animal or body-parts. Indeed, our own studies showed that very different sets of sex-differentially expressed genes are identified when whole animals, heads, or dissected CNS tissues are examined [26]–[27] and results in both flies and mice [4], [28] suggest that gene expression varies dramatically in different cell-types within a tissue. In future work, it will be interesting to determine how sensitive the TRAP approach is, with respect to the number of cells that can be accurately profiled, as well as how effective the technique is in detecting differences in the translatome in response to physiological and genetic perturbations. In sum, this method is versatile, simple, rapid, reproducible and amenable for high-throughput targeted translatome analysis under almost any condition.

## Materials and Methods

### Fly Stocks and Rearing Conditions

The following fly strains were obtained from the Bloomington stock center: *Elav-GAL4* [15], *50Y-GAL4* [22] and *UAS-eGFP*. The *UAS-GFP::RpL10A* stocks were generated by P-element transformation with the *pUAST* vector into which we directionally cloned *eGFP* cDNA in frame to the *Drosophila RpL10A* gene (**Figure S1**). The wild type flies were the Canton S strain. Transformants were generated as previously described [29]. All flies were reared on yeast, molasses and agar food at room temperature (22.5±0.5°C) on a 12-h light/12-h dark cycle.

### Polysome Immunoprecipitation

Approximately 500–1,000 heads per genotype from 7-day post eclosion flies of the appropriate genotype were collected and homogenized in extraction buffer (20 mM HEPES, pH 7.5, 150 mM KCl, 5 mM MgCl_2_, 1% Triton X-100, 0.5 mM DTT, 100 µg/mL Cyclohexamide, 100 U/mL RNase OUT, 1X Complete Protease Inhibitor (Roche)). Lysate was centrifuged to separate insoluble material and protein extract was added to Protein A Sepharose beads conjugated to mouse anti-GFP (NeuroMab N86/38). Lysate-bead slurry was incubated overnight at 4°C followed by washing in Wash Buffer (150 mM NaCl, 0.05% Triton X-100, 50 mM Tris, 5 mM MgCl_2_, and 40 U/mL RNase OUT) at 4°C. RNA was extracted using standard Trizol extraction methods for downstream analysis.

### Western Blotting

Protein was extracted from 300 heads in Extraction buffer (20 mM HEPES, pH 7.5, 100 mM KCl, 0.1% Triton X-100, 10 mM EDTA, 1 mM DTT, 5% glycerol, and Complete Protease Inhibitors (Roche)). Lysate was centrifuged at 4°C, supernatant was removed, and 30 µg was run on 8% polyacrylamide gel and transferred to nitrocellulose. The following antibodies were used according to the manufactures instructions; αGFP (Invitrogen #A11122 and NeuroMab #N86/38), αRpL10 (Sigma; #SAB1101199, and Abgent; #AT3701a), αRpS6 (Cell Signaling; #2317), αMouse-HRP (MP, #0855563), αRabbit-HRP (MP, #0855689).

### Polysome Sucrose Gradients

Heads from 300 flies were homogenized in 1xPBS containing 100 µg/mL cyclohexamide and 1% NP40. Samples were left on ice for 15 minutes followed by three differential centrifugation steps at 4°C (8,600×g, 5 min; 13,000×g, 5 min; 20,000×g, 10 min). Sucrose gradients were run as previously described [16]. Protein was extracted from each fraction by precipitation with Trichloroacetic acid followed by Acetone wash, and examined by western blotting.

### Quantitative RT-PCR

500 ng-1 µg of total RNA was used to generate cDNA using SuperScript III First-Strand Synthesis Kit and Oligo dT primer. Primers and probes were designed for *dIlp2* (F-CTCTGCAGTGAAAAGCTCAAC, R- CTCGAACTCCTGGACAAACTG, P- CTCGCACACCATACTCAGC), *NPF* (F-ACTCCCAGTTGAACCAGAAC, R- TCAGCCATAGTGTTGACATCG, P- CCAACTCCAGACCTCCGCG) and *β-Actin* (F- CCTCGAAATCGTAGCTCTACAC, R- ACCAGCCTGACCAACATG, P- TCACACGCGACAAGGAAAATT) from Integrated DNA Technologies. These primer/probe sets were used to quantify expression. Results were obtained and quantified using Applied Biosystems 7900 machine and SDS 2.3 software.

### Immunohistochemistry and Microscopy

For immunohistochemistry, adult brains were dissected in ice cold 4% PFA-PBS and were further fixed for a total of 60 min. Next, the brains were rinsed quickly 3 times with PBS-0.5% Triton X-100 (PBT) and then washed three times for 20 min in PBT at room temperature. The brains were then blocked in 5% normal goat serum-PBT for one hour at room temperature. Samples were incubated in 5% normal goat serum-PBT with primary antibody for two nights at 4°C. After three 20 min washes with PBT, the brains were incubated in 5% normal goat serum-PBT with secondary antibody for two nights at 4°C. The brains were then washed four times for 20 min and then overnight at 4°C. Finally, brains were mounted in SlowFade mounting medium (Invitrogen) and covered with a no. 0 glass coverslip. The immunostained brains were imaged with an inverted Zeiss Confocal Microscope (Axiovert 100 M). The following primary antibodies were used for immunofluoresence: mouse anti-Dlg (1∶100; Developmental Studies Hybridoma Bank); rabbit anti-GFP (1∶100; Invitrogen) or mouse anti-GFP (1∶200; NeuroMab). Live images were obtained from freshly dissected adult brains in ice cold 1xPBS and mounted in 1xPBS and imaged on a Zeiss Axioplan2 with an Apotome module using Axiovision software. Images were further processed with Adobe Photoshop.

### RNA Extraction, Library Preparation, Sequencing and Data Analysis

Total RNA from the polysome-enriched fraction and from whole heads was extracted using Trizol, and the polyA mRNA was purified using MicroPoly(A) Purist columns (Ambion). For the polysome purification, we started with ∼3 µg of total RNA and obtained ∼40 ng of polyA mRNA. For each polysome-enriched biological replicate ∼40 ng of mRNA was used for library construction. All subsequent steps of the Illumina library preparation were performed as previously described [17]. The libraries were sequenced on a single end, using an Illumina Genome Analyzer IIx sequencer, with 72 bases determined. The sequence reads were aligned to the annotated *Drosophila* genome and statistically analyzed, as previously described [30]. The polysome fraction libraries were made from three independent biological samples from roughly equal numbers of a mixture of male and female heads, whereas the whole head mRNA libraries were from separated male and female heads, three each. To identify transcripts that were enriched or depleted in the polysome fraction, as compared to whole head mRNA the male and female whole head samples were treated as one treatment group (6 independent replicates, 3 male and 3 female), and the polysome enriched fraction was treated as the other treatment group (3 independent replicates). To ensure that the differences in replicate number did not bias our results (6 versus 3 replicates), Illumina sequence reads from the male and female samples were pooled to generate 3 sets of independent whole head data (male and female mixed) and the data was statistically analyzed. We did not detect major differences in the results (data not shown).

## Supporting Information

Figure S1 **Generation of** ***GFP*** **tagged** ***RpL10Ab*** **fusion gene in** ***pUAST*** **vector.** We cloned *eGFP* sequences in frame and upstream of the *Drosophila melanogaster RpL10Ab* gene into the *Eco*RI-*Xho*I sites of the MCS of the *pUAST* vector. Cloning was performed in two steps as there is an endogenous *Xho*I site in *RpL10Ab*. The correct sequence of the fusion gene construct was verified by Sanger sequencing before P-element-based transformation. The use of tagged ribosomal proteins for translatome profiling has also been performed in other species [9]–[14].
(DOC)

Figure S2 **Perinuclear and nucleolar localization of GFP::RpL10A.** The panel on the left shows *Elav-GAL4>UAS-GFP::RpL10A* fly brain stained with antibodies against GFP (green) and Elav (red). Elav staining in the optic lobes is much stronger than GFP because the *Elav-GAL4* driver expresses at relatively low levels in these neurons. The panels on the right are a digitally magnified view of the white square in the left panel. The top right panel shows the merge and the panels below show GFP and Elav staining respectively. The GFP staining pattern recapitulates the strong perinuclear and nucleolar (white arrow) localization pattern of endogenous RpL10A.
(DOC)

Figure S3 **Quantification of GFP::RpL10A incorporation into ribosomes.** Sucrose gradient of head extracts from 300 *ElavGAL4>UAS-GFP::RpL10A* flies (as in Fig. 2A). Protein from each fraction was extracted and run on western blots that were probed with antibodies against GFP, RpS6 and RpL10 to detect the GFP::RpL10A fusion protein, endogenous RpS6 and RpL10 respectively (RpL10 antibody also detected a non-specific band that masked the fusion protein so that we could not use it to directly compare the signals in the GFP and RpL10 rows). In the table to the right of the figure we quantified the percentage of signal in each GFP lane compared to the signal in the corresponding RpS6 and RpL10 lanes, as a measure of incorporation of the fusion protein into each polysome fraction. The signal was normalized to the signal strength of each antibody in the input fraction to the left of the blots. Using this measure, the signal or incorporation of the GFP::RpL10A fusion protein in each polysome fraction varied between 11.8 and 34.9% when using RpL10 as the standard and between 21.8 and 72.6% when using RpS6 as the standard. On average the fusion protein incorporated almost 1/3 to 1/2 as well into polysomes as the endogenous proteins (average taken from all the values in the polysome fractions 5 to 14, marked in blue in the table).
(DOC)

Figure S4 **Localization of GFP::RpL10A in the** ***pars intercerebralis*** **.** Immunofluorescent image of the brain from a *50Y>UAS-GFP::RpL10A* male expressing GFP-tagged RpL10A in the *pars intercerebralis* (*PI*). GFP is marked in green and neuropil is marked with Dlg in red. The majority of the GFP positive cells are expressed in the *PI*, a group of approximately 200 neurosecretory cells in the dorsomedial protocerebrum (arrow head). In addition, a few GFP positive cells can be detected in the subesophageal ganglia and the dorsal protocerebrum.
(DOC)

Table S1 **Insertions of** ***UAS-GFP::RpL10A*** **lines.**
(DOC)

Table S2 **Neuronal translatome compared to whole head mRNA expression.** Raw RNA sequencing data (see attached Excel sheet). Data were analyzed as previously published [30]. As described in detail in the methods section, three independent replicates of mRNA extracted from heads of *CantonS* females (DM_CSF1, DM_CSF2, DM_CSF3), *CantonS* males (DM_CSM1, DM_CSM2, DM_CSM3) and polysome affinity purified *Elav-GAL4>UAS-GFP::RpL10A* males and females (DM_NT1, DM_NT2, DM_NT3) were sequenced on the Illumina platform.
(DOC)

Table S3 **Genes with transcripts enriched in glia are generally depleted from** ***Elav-GAL4>UAS-GFP::RpL10A*** **polysome preparations.** All the genes highlighted in blue have transcripts that are significantly depleted in neurons compared to whole heads. Genes not highlighted have transcripts that are unchanged and genes highlighted in red have transcripts that are significantly enriched in neurons compared to heads.
(DOC)

Table S4 **Genes with transcripts enriched in fat body are generally depleted from** ***Elav-GAL4>UAS-GFP::RpL10A*** **polysome preparations.** All the genes highlighted in blue have transcripts that are significantly depleted in neurons compared to heads. Genes not highlighted have transcripts that are unchanged and genes highlighted in red have transcripts that are significantly enriched in neurons compared to heads.
(DOC)

Table S5 **Genes with transcripts enriched in the cuticle are generally depleted from** ***Elav-GAL4>UAS-GFP::RpL10A*** **polysome preparations.** All the genes highlighted in blue have transcripts that are significantly depleted in neurons compared to heads. Genes not highlighted have transcripts that are unchanged and genes highlighted in red have transcripts that are significantly enriched in neurons compared to heads. Overall, 57% of the genes with predicted expression in glia [18], fat body [19] and cuticle [20] have transcripts that are significantly depleted in neurons, 16% are enriched and 27% are not significantly changed.
(DOC)
