# Correction: A Versatile Method for Cell-Specific Profiling of Translated mRNAs in *Drosophila*

**DOI:** 10.1371/annotation/39194a57-4480-4f8e-b6fa-e7e0993d029b

**Published:** 2013-07-24

**Authors:** Amanda Thomas, Pei-Jung Lee, Justin E. Dalton, Krystle J. Nomie, Loredana Stoica, Mauro Costa-Mattioli, Peter Chang, Sergey Nuzhdin, Michelle N. Arbeitman, Herman A. Dierick

In the Supporting Information section, the wrong file was given as Table S2. Please see the file at the following link, which is the correct version of Table S2 (Excel spreadsheet): http://plosone.org/corrections/pone.0040276.s002.cn.xls

