# Supplementary material for: A Versatile Method for Cell-Specific Profiling of Translated mRNAs in Drosophila
Source: PLoS One. 2012 Jul 6;7(7):e40276. doi: 10.1371/journal.pone.0040276 (PMC3391276; doi:10.1371/journal.pone.0040276)
Supplement: Figure S1 — Generation of GFP tagged RpL10Ab fusion gene in pUAST vector. We cloned eGFP sequences in frame and upstream of the Drosophila melanogaster RpL10Ab gene into the EcoRI-XhoI sites of the MCS of the pUAST vector. Cloning was performed in two steps as there is an endogenous XhoI site in RpL10Ab. The correct sequence of the fusion gene construct was verified by Sanger sequencing before P-element-based transformation. The use of tagged ribosomal proteins for translatome profiling has also been performed in other species [9]–[14]. (DOC) [file pone.0040276.s001.doc]

Figure S1. Generation of *GFP* tagged *RpL10Ab* fusion gene in *pUAST* vector.


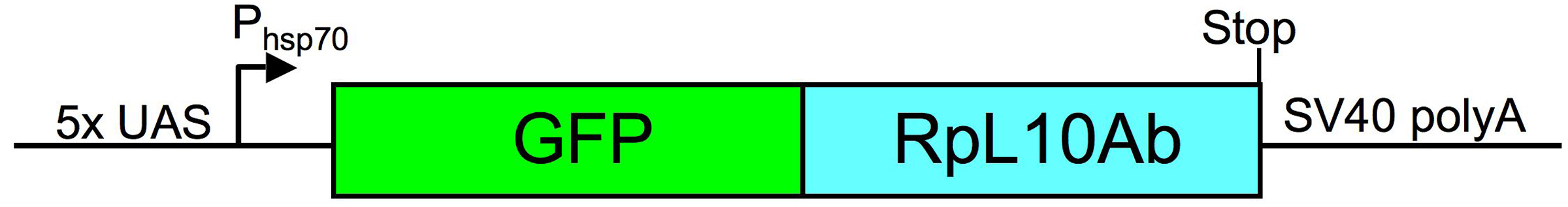


We cloned *eGFP* sequences in frame and upstream of the *Drosophila melanogaster* *RpL10Ab* gene into the *Eco*RI-*Xho*I sites of the MCS of the *pUAST* vector. Cloning was performed in two steps as there is an endogenous *Xho*I site in *RpL10Ab*. The correct sequence of the fusion gene construct was verified by Sanger sequencing before P-element-based transformation. The use of tagged ribosomal proteins for translatome profiling has also been performed in other species [9-14].
