# Supplementary material for: A Versatile Method for Cell-Specific Profiling of Translated mRNAs in Drosophila
Source: PLoS One. 2012 Jul 6;7(7):e40276. doi: 10.1371/journal.pone.0040276 (PMC3391276; doi:10.1371/journal.pone.0040276)
Supplement: Figure S2 — Perinuclear and nucleolar localization of GFP::RpL10A. The panel on the left shows Elav-GAL4>UAS-GFP::RpL10A fly brain stained with antibodies against GFP (green) and Elav (red). Elav staining in the optic lobes is much stronger than GFP because the Elav-GAL4 driver expresses at relatively low levels in these neurons. The panels on the right are a digitally magnified view of the white square in the left panel. The top right panel shows the merge and the panels below show GFP and Elav staining respectively. The GFP staining pattern recapitulates the strong perinuclear and nucleolar (white arrow) localization pattern of endogenous RpL10A. (DOC) [file pone.0040276.s002.doc]

Figure S2. Perinuclear and nucleolar localization of GFP::RpL10A.


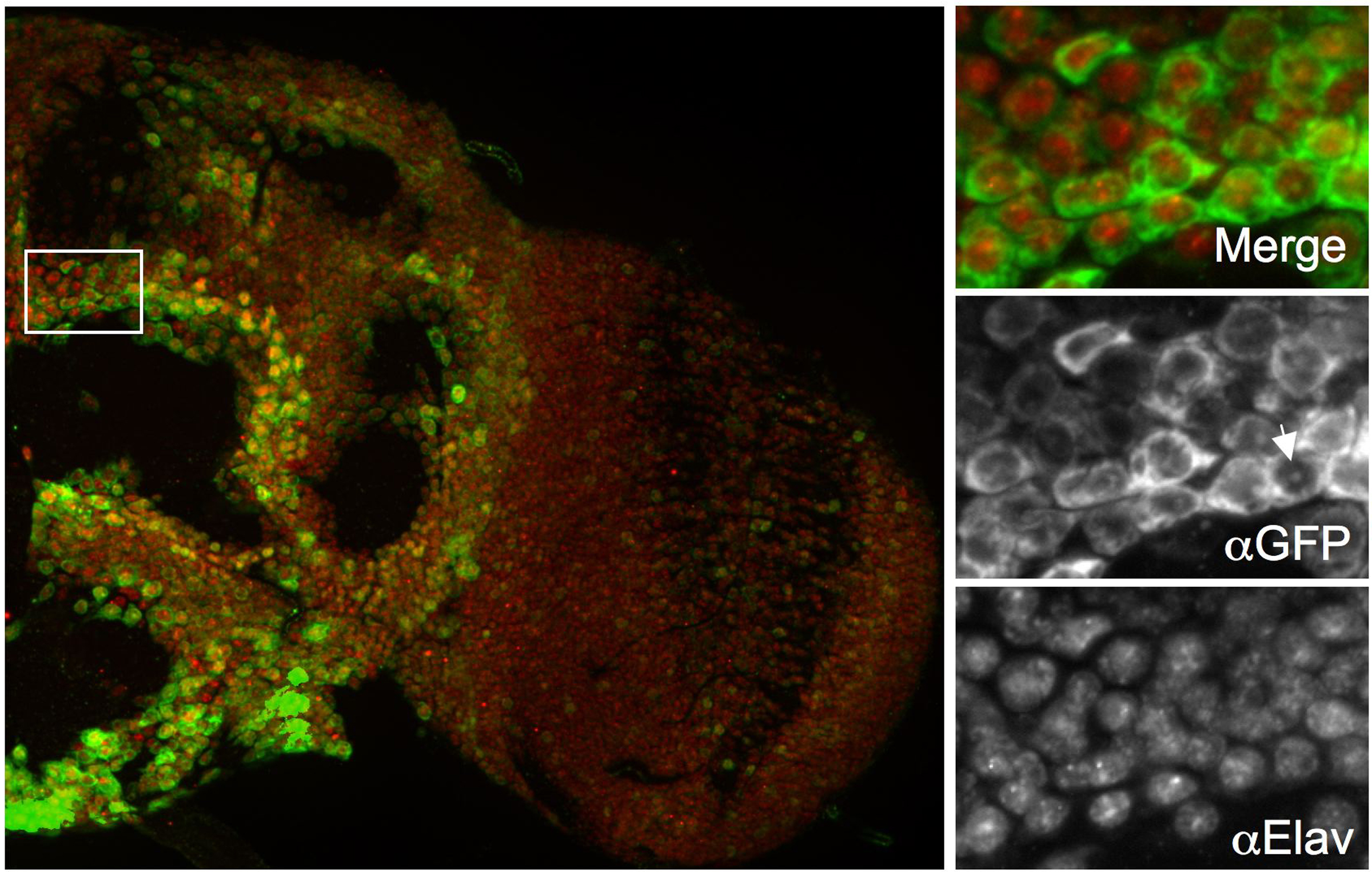


The panel on the left shows *Elav-GAL4>UAS-GFP::RpL10A* fly brain stained with antibodies against GFP (green) and Elav (red). Elav staining in the optic lobes is much stronger than GFP because the *Elav-GAL4* driver expresses at relatively low levels in these neurons. The panels on the right are a digitally magnified view of the white square in the left panel. The top right panel shows the merge and the panels below show GFP and Elav staining respectively. The GFP staining pattern recapitulates the strong perinuclear and nucleolar (white arrow) localization pattern of endogenous RpL10A.
