# Supplementary material for: A Versatile Method for Cell-Specific Profiling of Translated mRNAs in Drosophila
Source: PLoS One. 2012 Jul 6;7(7):e40276. doi: 10.1371/journal.pone.0040276 (PMC3391276; doi:10.1371/journal.pone.0040276)
Supplement: Figure S3 — Quantification of GFP::RpL10A incorporation into ribosomes. Sucrose gradient of head extracts from 300 ElavGAL4>UAS-GFP::RpL10A flies (as in Fig. 2A). Protein from each fraction was extracted and run on western blots that were probed with antibodies against GFP, RpS6 and RpL10 to detect the GFP::RpL10A fusion protein, endogenous RpS6 and RpL10 respectively (RpL10 antibody also detected a non-specific band that masked the fusion protein so that we could not use it to directly compare the signals in the GFP and RpL10 rows). In the table to the right of the figure we quantified the percentage of signal in each GFP lane compared to the signal in the corresponding RpS6 and RpL10 lanes, as a measure of incorporation of the fusion protein into each polysome fraction. The signal was normalized to the signal strength of each antibody in the input fraction to the left of the blots. Using this measure, the signal or incorporation of the GFP::RpL10A fusion protein in each polysome fraction varied between 11.8 and 34.9% when using RpL10 as the standard and between 21.8 and 72.6% when using RpS6 as the standard. On average the fusion protein incorporated almost 1/3 to 1/2 as well into polysomes as the endogenous proteins (average taken from all the values in the polysome fractions 5 to 14, marked in blue in the table). (DOC) [file pone.0040276.s003.doc]

Figure S3. Quantification of GFP::RpL10A incorporation into ribosomes.


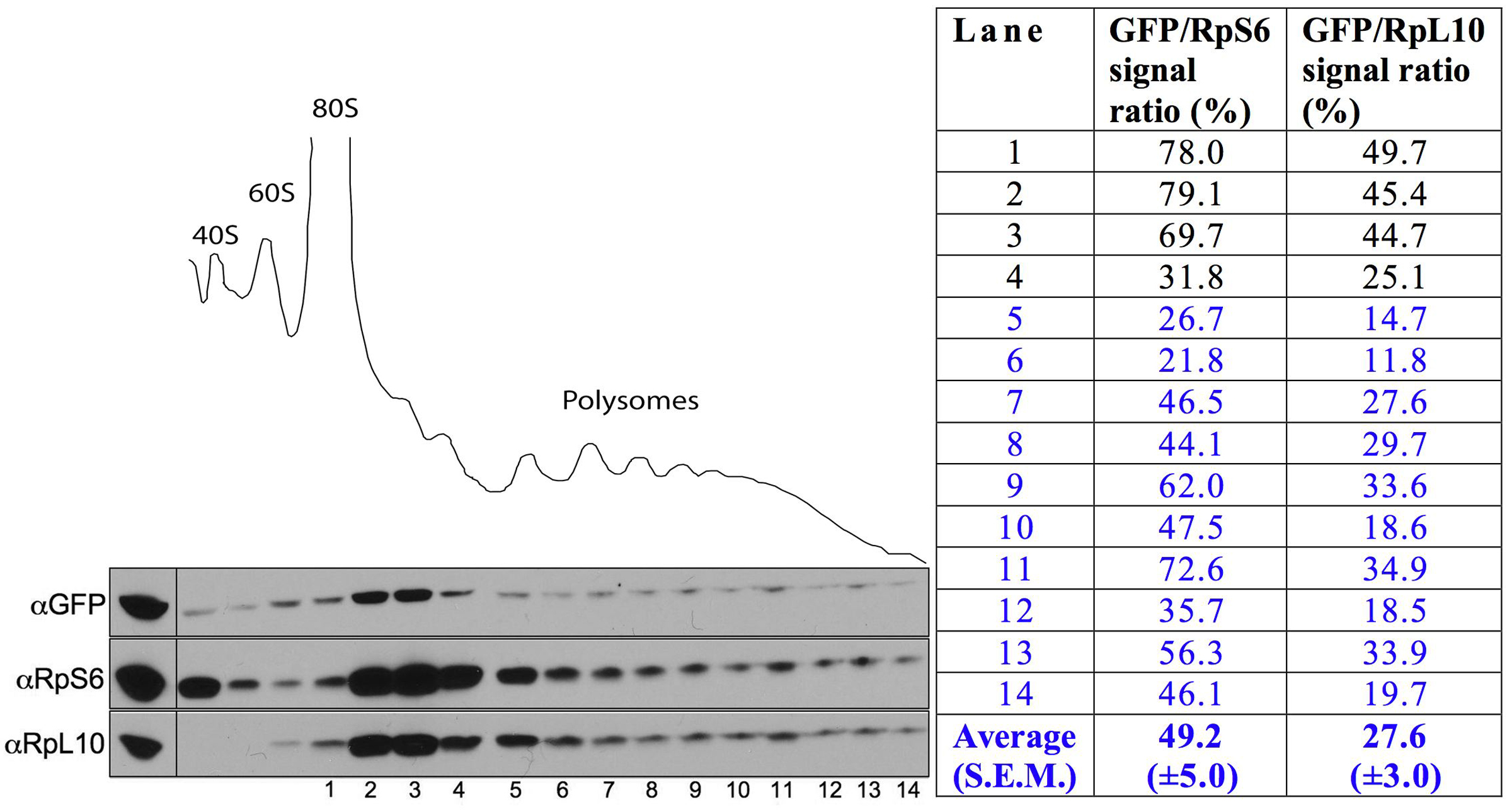


Sucrose gradient of head extracts from 300 *ElavGAL4>UAS-GFP::RpL10A* flies (as in Fig. 2A). Protein from each fraction was extracted and run on western blots that were probed with antibodies against GFP, RpS6 and RpL10 to detect the GFP::RpL10A fusion protein, endogenous RpS6 and RpL10 respectively (RpL10 antibody also detected a non-specific band that masked the fusion protein so that we could not use it to directly compare the signals in the GFP and RpL10 rows). In the table to the right of the figure we quantified the percentage of signal in each GFP lane compared to the signal in the corresponding RpS6 and RpL10 lanes, as a measure of incorporation of the fusion protein into each polysome fraction. The signal was normalized to the signal strength of each antibody in the input fraction to the left of the blots. Using this measure, the signal or incorporation of the GFP::RpL10A fusion protein in each polysome fraction varied between 11.8 and 34.9 % when using RpL10 as the standard and between 21.8 and 72.6 % when using RpS6 as the standard. On average the fusion protein incorporated almost 1/3 to 1/2 as well into polysomes as the endogenous proteins (average taken from all the values in the polysome fractions 5 to 14, marked in blue in the table).
