# Supplementary material for: A Versatile Method for Cell-Specific Profiling of Translated mRNAs in Drosophila
Source: PLoS One. 2012 Jul 6;7(7):e40276. doi: 10.1371/journal.pone.0040276 (PMC3391276; doi:10.1371/journal.pone.0040276)
Supplement: Figure S4 — Localization of GFP::RpL10A in the pars intercerebralis . Immunofluorescent image of the brain from a 50Y>UAS-GFP::RpL10A male expressing GFP-tagged RpL10A in the pars intercerebralis (PI). GFP is marked in green and neuropil is marked with Dlg in red. The majority of the GFP positive cells are expressed in the PI, a group of approximately 200 neurosecretory cells in the dorsomedial protocerebrum (arrow head). In addition, a few GFP positive cells can be detected in the subesophageal ganglia and the dorsal protocerebrum. (DOC) [file pone.0040276.s004.doc]

Figure S4. Localization of GFP::RpL10A in the *pars intercerebralis*.


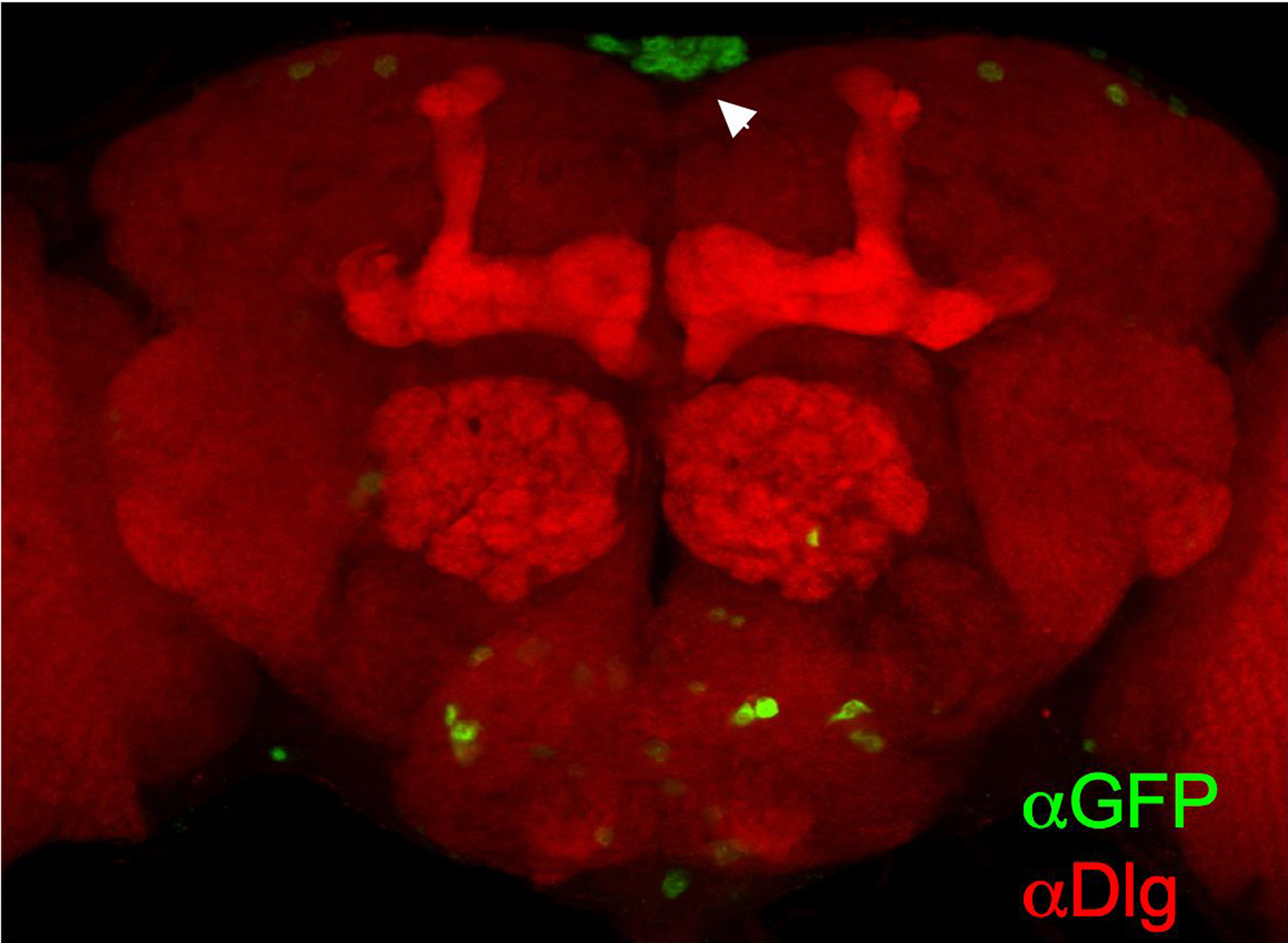


Immunofluorescent image of the brain from a *50Y>UAS-GFP::RpL10A* male expressing GFP-tagged RpL10A in the *pars intercerebralis* (*PI*). GFP is marked in green and neuropil is marked with Dlg in red. The majority of the GFP positive cells are expressed in the *PI*, a group of approximately 200 neurosecretory cells in the dorsomedial protocerebrum (arrow head). In addition, a few GFP positive cells can be detected in the subesophageal ganglia and the dorsal protocerebrum.
