# Supplementary material for: A Versatile Method for Cell-Specific Profiling of Translated mRNAs in Drosophila
Source: PLoS One. 2012 Jul 6;7(7):e40276. doi: 10.1371/journal.pone.0040276 (PMC3391276; doi:10.1371/journal.pone.0040276)
Supplement: Table S1 — Insertions of UAS-GFP::RpL10A lines. (DOC) [file pone.0040276.s005.doc]

Table S1. Insertions of *UAS-GFP::RpL10A* lines.

| **Name of Insertion** | **Chrom** | **Recessive Lethality** | **Lethality upon ubiquitous expression (*Act5C-GAL4*)** | **Lethality upon pan-neuronal expression**  **(*Elav-GAL4*)** | **Expression level with *Elav-GAL4* driver** |
| --- | --- | --- | --- | --- | --- |
| BF2 | 3 | No | No | No | ++++ |
| BF2b | 2 | No | No | No | ++++ |
| BF3 | 2 | No | Pupal lethal with few escapers | No | +++ |
| BF10 | 3 | No | No | No | +++ |
| BF12 | 2 | No | Pupal lethal with few escapers | No | ++ |
| BF14 | 2 | No | No | No | ++++ |
| BF19 | X | No | Pupal lethal with few escapers | Yes | ++++ |
| BF24 | X | No | No | No | ++++ |
| BF27 | 3 | No | No | No | ++++ |
| BF28 | 2 | No | No | No | + |
| BM3 | 3 | No | No | No | ++ |
| BM10 | 3 | No | NA | No | ++ |
| BM11 | 2 | Yes | Yes | No | ++++ |
| BM15 | 3 | No | Pupal lethal with few escapers | No | ++ |
| BM16 | ? | No | Pupal lethal with few escapers | No | +++ |
| BM23 | 3 | Yes | No | No | +++ |
