# Supplementary material for: A Versatile Method for Cell-Specific Profiling of Translated mRNAs in Drosophila
Source: PLoS One. 2012 Jul 6;7(7):e40276. doi: 10.1371/journal.pone.0040276 (PMC3391276; doi:10.1371/journal.pone.0040276)
Supplement: Table S2 — Neuronal translatome compared to whole head mRNA expression. Raw RNA sequencing data (see attached Excel sheet). Data were analyzed as previously published [30]. As described in detail in the methods section, three independent replicates of mRNA extracted from heads of CantonS females (DM_CSF1, DM_CSF2, DM_CSF3), CantonS males (DM_CSM1, DM_CSM2, DM_CSM3) and polysome affinity purified Elav-GAL4>UAS-GFP::RpL10A males and females (DM_NT1, DM_NT2, DM_NT3) were sequenced on the Illumina platform. (DOC) [file pone.0040276.s006.doc]

Table S2. Neuronal translatome compared to whole head mRNA expression.

Raw RNA sequencing data (see attached Excel sheet). Data were analyzed as previously published [30]. As described in detail in the methods section, three independent replicates of mRNA extracted from heads of *CantonS* females (DM_CSF1, DM_CSF2, DM_CSF3), *CantonS* males (DM_CSM1, DM_CSM2, DM_CSM3) and polysome affinity purified *Elav-GAL4>UAS-GFP::RpL10A* males and females (DM_NT1, DM_NT2, DM_NT3) were sequenced on the Illumina platform.
