# Supplementary material for: A Versatile Method for Cell-Specific Profiling of Translated mRNAs in Drosophila
Source: PLoS One. 2012 Jul 6;7(7):e40276. doi: 10.1371/journal.pone.0040276 (PMC3391276; doi:10.1371/journal.pone.0040276)
Supplement: Table S3 — Genes with transcripts enriched in glia are generally depleted from Elav-GAL4>UAS-GFP::RpL10A polysome preparations. All the genes highlighted in blue have transcripts that are significantly depleted in neurons compared to whole heads. Genes not highlighted have transcripts that are unchanged and genes highlighted in red have transcripts that are significantly enriched in neurons compared to heads. (DOC) [file pone.0040276.s007.doc]

Table S3. Genes with transcripts enriched in glia are generally depleted from *Elav-GAL4>UAS-GFP::RpL10A* polysome preparations.

| **FlyBase ID** | **Gene Name** | **Chrom** | **Whole Head Mean (Fkpm)** | **SEM** | **RpL10A IP Mean (Fkpm)** | **SEM** | **Fold Depletion** |
| --- | --- | --- | --- | --- | --- | --- | --- |
| FBgn0016075 | *vkg* | 2L | 367.00 | 14.79 | 22.12 | 0.82 | 16.59 |
| FBgn0014011 | *Rac2* | 3L | 48.05 | 1.29 | 4.95 | 0.02 | 9.70 |
| FBgn0261546 | *axo* | 3L | 75.73 | 4.88 | 9.57 | 0.29 | 7.91 |
| FBgn0051235 | *CG31235* | 3R | 16.78 | 2.83 | 2.31 | 0.34 | 7.26 |
| FBgn0026585 | *Ent2* | 2L | 20.93 | 0.84 | 2.98 | 1.01 | 7.03 |
| FBgn0010389 | *htl* | 3R | 22.14 | 2.31 | 3.96 | 0.02 | 5.59 |
| FBgn0026439 | *Eaat1* | 2L | 550.54 | 27.60 | 99.05 | 1.09 | 5.56 |
| FBgn0015777 | *nrv2* | 2L | 570.17 | 34.71 | 113.21 | 5.47 | 5.04 |
| FBgn0003118 | *pnt* | 3R | 18.58 | 1.10 | 3.96 | 0.02 | 4.69 |
| FBgn0003507 | *srp* | 3R | 60.16 | 6.18 | 12.88 | 0.55 | 4.67 |
| FBgn0011701 | *repo* | 3R | 38.53 | 2.17 | 8.59 | 0.90 | 4.49 |
| FBgn0035371 | *CG9977* | 3L | 60.12 | 2.28 | 14.86 | 2.00 | 4.05 |
| FBgn0025878 | *wrapper* | 2R | 29.52 | 3.60 | 7.60 | 0.70 | 3.89 |
| FBgn0027932 | *Akap200* | 2L | 386.29 | 56.44 | 102.38 | 7.99 | 3.77 |
| FBgn0037207 | *Mes2* | 3L | 13.41 | 0.91 | 3.63 | 0.66 | 3.69 |
| FBgn0004513 | *Mdr65* | 3L | 166.81 | 3.64 | 45.89 | 0.47 | 3.63 |
| FBgn0011746 | *ana* | 2R | 17.59 | 1.17 | 4.95 | 0.56 | 3.55 |
| FBgn0027594 | *drpr* | 3L | 144.00 | 3.41 | 56.12 | 1.49 | 2.57 |
| FBgn0002968 | *Nrg* | X | 268.09 | 16.73 | 106.12 | 26.12 | 2.53 |
| FBgn0028479 | *CG4389* | 2L | 147.66 | 9.12 | 64.39 | 1.57 | 2.29 |
| FBgn0013770 | *Cp1* | 2R | 844.50 | 46.85 | 369.16 | 8.15 | 2.29 |
| FBgn0043841 | *vir-1* | 2L | 288.79 | 14.64 | 132.05 | 2.81 | 2.19 |
| FBgn0038975 | *Nrx-1* | 3R | 224.44 | 19.52 | 108.69 | 11.26 | 2.06 |
| FBgn0003870 | *ttk* | 3R | 110.02 | 7.59 | 54.81 | 1.32 | 2.01 |
| FBgn0025631 | *moody* | X | 77.24 | 2.90 | 39.60 | 3.25 | 1.95 |
| FBgn0031745 | *CG8965* | 2L | 5.19 | 0.76 | 2.97 | 0.58 | 1.75 |
| FBgn0261673 | *nemy* | 2R | 43.77 | 4.14 | 25.42 | 0.66 | 1.72 |
| FBgn0028647 | *CG11902* | 3R | 27.47 | 1.24 | 16.50 | 1.36 | 1.66 |
| FBgn0000416 | *Sap-r* | 3R | 973.34 | 27.44 | 621.32 | 13.83 | 1.57 |
| FBgn0020278 | *loco* | 3R | 33.48 | 2.44 | 22.77 | 1.41 | 1.47 |
| FBgn0015776 | *nrv1* | 2L | 248.48 | 7.89 | 170.37 | 8.81 | 1.46 |
| FBgn0000157 | *Dll* | 2R | 15.41 | 0.50 | 11.22 | 1.74 | 1.37 |
| FBgn0010238 | *Lac* | 2R | 102.15 | 0.81 | 84.85 | 1.34 | 1.20 |
| FBgn0000116 | *Argk* | 3L | 1502.25 | 103.10 | 1302.88 | 43.54 | 1.15 |
| FBgn0001145 | *Gs2* | X | 1131.52 | 66.94 | 1020.64 | 1.96 | 1.11 |
| FBgn0026438 | *Eaat2* | 2L | 325.98 | 19.12 | 302.39 | 11.24 | 1.08 |
| FBgn0050342 | *Prp38* | 2R | 12.40 | 0.57 | 11.88 | 0.52 | 1.04 |
| FBgn0039665 | *CG2310* | 3R | 6.53 | 0.76 | 6.27 | 0.64 | 1.04 |
| FBgn0013763 | *Chit* | 2R | 235.12 | 8.38 | 241.58 | 16.08 | 0.97 |
| FBgn0038321 | *CG6218* | 3R | 60.38 | 5.69 | 66.71 | 2.13 | 0.91 |
| FBgn0034013 | *unc-5* | 2R | 44.72 | 1.30 | 52.17 | 0.68 | 0.86 |
| FBgn0004795 | *retn* | 2R | 47.68 | 1.77 | 55.81 | 1.54 | 0.85 |
| FBgn0052423 | *shep* | 3L | 474.23 | 40.06 | 638.99 | 23.57 | 0.74 |
| FBgn0031590 | *CG3702* | 2L | 73.62 | 6.80 | 104.36 | 3.06 | 0.71 |
| FBgn0034136 | *DAT* | 2R | 22.63 | 1.97 | 40.60 | 2.32 | 0.56 |
| FBgn0261285 | *Ppcs* | 3R | 73.01 | 1.46 | 139.34 | 3.38 | 0.52 |
| FBgn0010774 | *Aly* | 3R | 46.76 | 2.94 | 89.49 | 2.32 | 0.52 |
| FBgn0028425 | *JhI-21* | 2L | 26.00 | 3.06 | 54.49 | 1.33 | 0.48 |
| FBgn0033502 | *CG12910* | 2R | 9.05 | 0.40 | 20.47 | 2.15 | 0.44 |
| FBgn0037913 | *CG6783* | 3R | 1133.03 | 80.70 | 2836.81 | 150.52 | 0.40 |

All the genes highlighted in blue have transcripts that are significantly depleted in neurons compared to whole heads. Genes not highlighted have transcripts that are unchanged and genes highlighted in red have transcripts that are significantly enriched in neurons compared to heads.
